# Exploring the Effects of Selenium and Brassinosteroids on Photosynthesis and Protein Expression Patterns in Tomato Plants under Low Temperatures

**DOI:** 10.3390/plants12193351

**Published:** 2023-09-22

**Authors:** Taiba Saeed, Tanveer Alam Khan, Aqeel Ahmad, Mohammad Yusuf, Sajeesh Kappachery, Qazi Fariduddin, Gaurav Mudgal, Mayank Anand Gururani

**Affiliations:** 1 Department of Biosciences, Integral University, Kursi Rd., Lucknow 226026, India; 2 Plant Biotechnology Section, Department of Botany, Faculty of Life Sciences, Aligarh Muslim University, Aligarh 202002, India; 3 Department of Biology, College of Science, United Arab Emirates University, Al Ain 15551, United Arab Emirates; 4 University of Chinese Academy of Sciences (UCAS), Beijing 100049, China; 5 Plant Physiology and Biochemistry Section, Department of Botany, Faculty of Life Sciences, Aligarh Muslim University, Aligarh 202002, India; 6 University Institute of Biotechnology, Chandigarh University, Mohali 140413, India

**Keywords:** antioxidants, brassinosteroids, low-temperature, proteomics, Se, 2-DE

## Abstract

This study aimed to assess the effects of low-temperature stress on two tomato cultivars (S-22 and PKM-1) treated with 24-epibrassinolide (EBL) and selenium (Se) by determining the changes in the proteomics profiles, growth biomarkers, biochemical parameters, and physiological functions. The growth parameters, photosynthetic traits, and activity of nitrate reductase in the S-22 and PKM-1 plants were markedly reduced by exposure to low temperatures. However, the combined application of EBL and Se under different modes significantly enhanced the aforementioned parameters under stress and non-stress conditions. Exposure to low temperatures increased the activities of the antioxidant enzymes (catalase, peroxidase, and superoxide dismutase) and the proline content of leaves, which were further enhanced by treatment with Se and EBL in both varieties. This research sheds light on the potential for employing exogenous EBL and Se as crucial biochemical tactics to assist tomato plants in surviving low-temperature stress. Moreover, the differentially expressed proteins that were involved in plant metabolism following the combined application of EBL and Se under low-temperature stress were additionally identified. Functional analysis revealed that the Q54YH4 protein plays an active role against plant stressors. The conserved regions in the protein sequences were analyzed for assessing the reliability of plant responses against the external application of EBL and Se under low temperatures.

## 1. Introduction

Plants are frequently exposed to diverse environmental stresses during their lifespan, which reduces crop yield. Low-temperature stress is one of the most severe abiotic stresses in horticulture crops and significantly limits their productivity [1,2,3]. Cold temperatures reduce the wilting of leaves, chlorosis, necrosis, cell membrane repair, and oxidative stress in plants [4]. Low temperatures alter cellular staying power, cell division, developmental performance, and cellular antioxidant activity, as well as water transport, which have deleterious effects on the yield [2,3,5]. Low-temperature stress also suppresses the photosynthetic capacity and efficacy by altering stomatal opening, gas exchange, production of pigment substances, and improving the functions of chloroplasts [2,3,6]. Low-temperature stress alters the metabolic cascade during metabolic development, which in turn alters the antioxidant system, location of membrane fatty acids, and gene regulation [7].

Se is an essential micronutrient in several organisms, including humans, and plays an important role in numerous plant species [8,9]. Se at toxic levels impairs the metabolism of ROS and MG, which exacerbates oxidative stress and causes plants to develop and produce less biomass [10]. In plants, selenate is absorbed via root sulfate transporters in the plasma membrane [10]. It has been reported that Se insufficiency or the presence of low quantities of Se leads to Se buildup in plant tissues [11]. Contrarily, Smith et al. [12] revealed that a reduction in gene expression leads to the insufficiency of sulfate transporters. However, the uptake of selenates may occur via compact contest in Se-deficient plants, which significantly augments the growth of vegetative and grain tissues [11]. It has been reported that minute concentrations of Se have positive effects in potato [13] and green algae [14]. The impact of Se on various plant species might change significantly depending on the stage of plant development, the length of exposure, as well as the shape, size, chemical composition, concentration, surface structure, and aggregation of the plants. A study on the impact of Se on *Hordeum vulgare* seeds revealed that Se has a favorable impact on the length of shoots, the size of roots, and the rate of germination. The sample treated with Se had the highest seed germination percentage [15]. Previous studies have demonstrated that Se helps in overcoming the stress response of plants by scavenging excess reactive oxygen species (ROS) via the enhancement of antioxidant enzymes and the production of alkaloids, flavonoids, ascorbate, and carotenoids [15,16,17]. Furthermore, the treatment of *Oryza sativa* with Se has been shown to have a positive effect on photosynthetic parameters, which improves the grain yield of rice [18]. Similar results have been observed in other crops treated with Se, including *Brassica juncea* [16,17], *Lolium perenne* [19], *Solanum tuberosum* [13], *B*. *rapa* [20], and *Lens culinaris* [21]. Numerous studies have demonstrated that Se can mitigate the deleterious effects of various abiotic stresses, including water deficiency [22], salinity [23], temperature [24], and heavy metal [16] stress, on plants.

Brassinosteroids (BRs) are a newly discovered class of polyhydroxy steroidal hormones of plants that act as endogenous signals and control plant growth and expansion [25,26]. BRs influence several typical physiological and developmental processes, including seed germination, blooming, and hypocotyl elongation [27]. However, BRs can ameliorate several abiotic stressors, including chills [28,29], metals [30,31,32], low temperatures [2,33,34], elevated temperatures [35], droughts [36,37], oxidative damage [38], and salinity [30,39,40]. The analog of BR, 24-epibrassinolide (EBL), confers plant protection under various unfavorable environmental cues, including heat stress, droughts, low temperatures, heavy metal stress, and salinity [2,6,33,34,41,42]. Therefore, the present study aimed to determine the effects of combined treatment with EBL (foliar administration) and Se (administered via the soil) on the protein expression, growth, biochemical factors, and physiological parameters of the leaves of two varieties of tomato (S-22 and PKM-1) under low-temperature stress.

## 2. Results

### 2.1. Growth Traits

**Figure 1:**
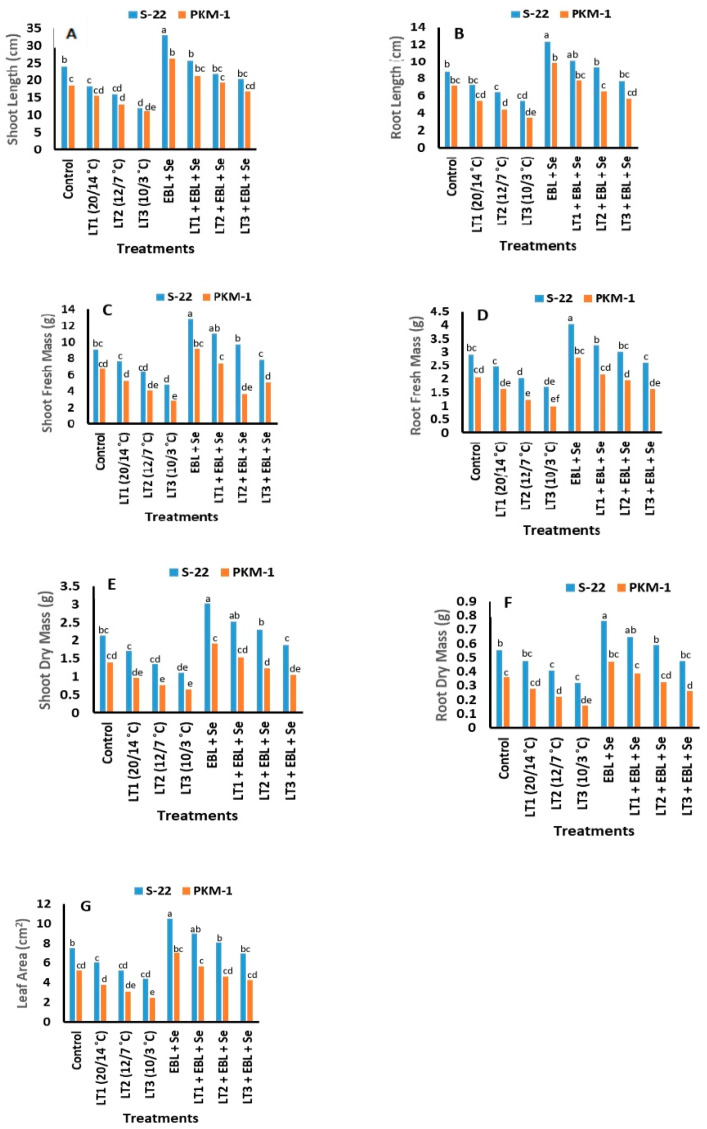
Effects of combined treatment with EBL and Se on the changes in (**A**) shoot length, (**B**) root length, (**C**) fresh mass of shoot, (**D**) fresh mass of root, (**E**) dry mass of shoot, (**F**) dry mass of root, and (**G**) leaf area in the S-22 and PKM-1 varieties of tomato, induced by low temperatures. All the data are the mean of five replicates (n = 5). Means not sharing the same letters above the bars are significantly different at *p* ≤ 0.05.

The increase in selected growth biomarkers, namely, the lengths of the shoots and roots, fresh and dry masses of the roots and shoots, and leaf areas, under low temperatures (20/14, 12/7, or 10/3 °C) was significantly lower than that at high temperatures, and this effect was more apparent in the PKM-1 variety than in S-22 plants. The negative effects were mostly observed in plants exposed to the lowest temperature of 10/3 °C. The combination of EBL and Se improved the shoot lengths, root lengths, dry mass of shoots, and dry mass of roots by 43%, 41%, 42%, and 39%, respectively, in S-22 plants, and by 38%, 35%, 32%, and 27%, respectively, in PKM-1 plants at 60 days after sowing (DAS; Figure 1A–G). The leaves of S-22 plants were fully exacerbated following exposure to all three levels of low-temperature stress (20/14, 12/7, and 10/3 °C); however, the leaves of PKM-1 plants were exacerbated following exposure to just two levels of low-temperature stress (20/14 and 12/7 °C). The other development traits also exhibited a similar pattern. However, treatment with EBL + Se completely reversed the damage caused by exposure to low temperatures of 20/14 and 12/7 °C.

### 2.2. SPAD Value of Chlorophyll

**Figure 2:**
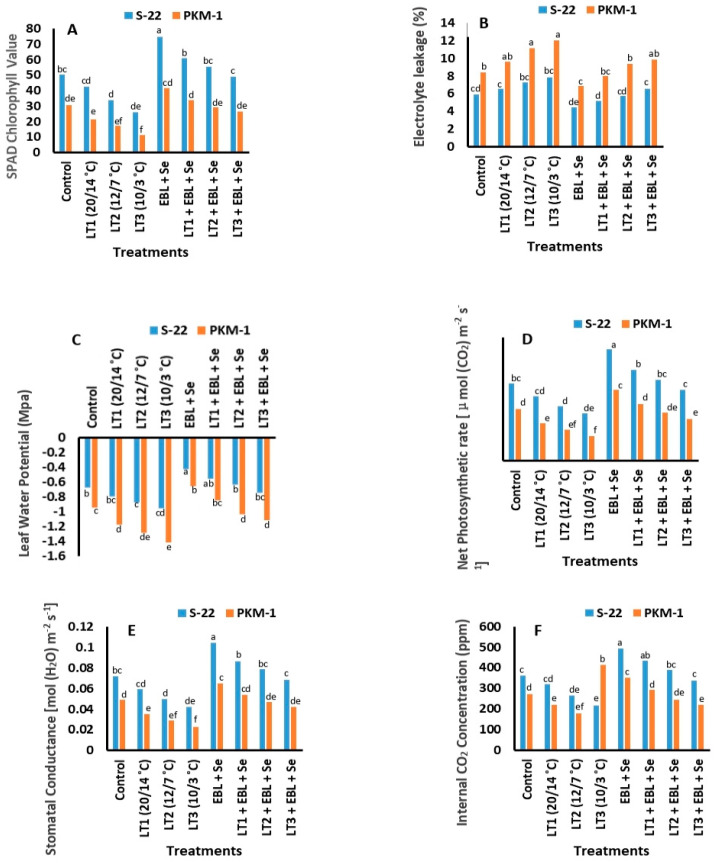
Combined treatment with EBL and Se altered the (**A**) SPAD chlorophyll levels, (**B**) electrolyte leakage, (**C**) leaf water potential, (**D**) net photosynthetic rate, (**E**) stomatal conductance, and (**F**) internal CO_2_ concentration of the S-22 and PKM-1 varieties of tomato at 60 DAS. All the data are the mean of five replicates (n = 5). Means not sharing the same letters above the bars are significantly different at *p* ≤ 0.05.

Compared to those of the control plants, the SPAD chlorophyll levels of S-22 and PKM-1 plants increased by 47% and 38%, respectively, following combined treatment with EBL and Se (Figure 2A) at 60 DAS. The SPAD chlorophyll levels of S-22 and PKM-1 plants exposed to the lowest temperature of 10/3 °C decreased markedly by 48% and 61%, respectively, at 60 DAS. Combined treatment with EBL + Se completely reversed the damage caused by exposure to the lowest temperature of 10/3 °C in both the varieties; however, the damage caused by exposure to temperatures of 12/7 °C was completely reversed by the application of EBL + Se in S-22 plants, but the reversal was moderate in PKM-1 plants.

### 2.3. Electrolyte Leakage

The plants exposed to low temperatures of 20/14, 12/7, or 10/3 °C exhibited increased electrolyte leakage. It was observed that ion leakage was highest in S-22 and PKM-1 plants exposed to the lowest temperature of 10/3 °C, being 32% and 42% higher, respectively, than that of the untreated plants at 60 DAS. Combined treatment with EBL and Se markedly reduced ion leakage at 60 DAS. The subsequent treatment of S-22 plants with EBL and Se completely reversed the damage caused by exposure to low temperatures of 20/14 and 12/7 °C; however, the combined treatment of PKM-1 plants only reversed the damage induced by exposure to the lowest temperature of 10/3 °C at 60 DAS (Figure 2B).

### 2.4. Leaf Water Potential (LWP)

The LWP was significantly reduced in both varieties of tomato (Figure 2C) following exposure to low-temperature stress. However, combined treatment with EBL + Se improved the LWP of S-22 and PKM-1 plants by 36% and 30%, respectively, compared to that of the respective control plants. The combined treatment of S-22 plants with EBL + Se completely reversed the reduction in LWP following exposure to all three levels of low-temperature stress; however, the combined treatment of PKM-1 plants with EBL + Se only improved the LWP at temperatures of 20/20 and 14/12 °C.

### 2.5. Photosynthesis and Associated Traits

**Figure 3:**
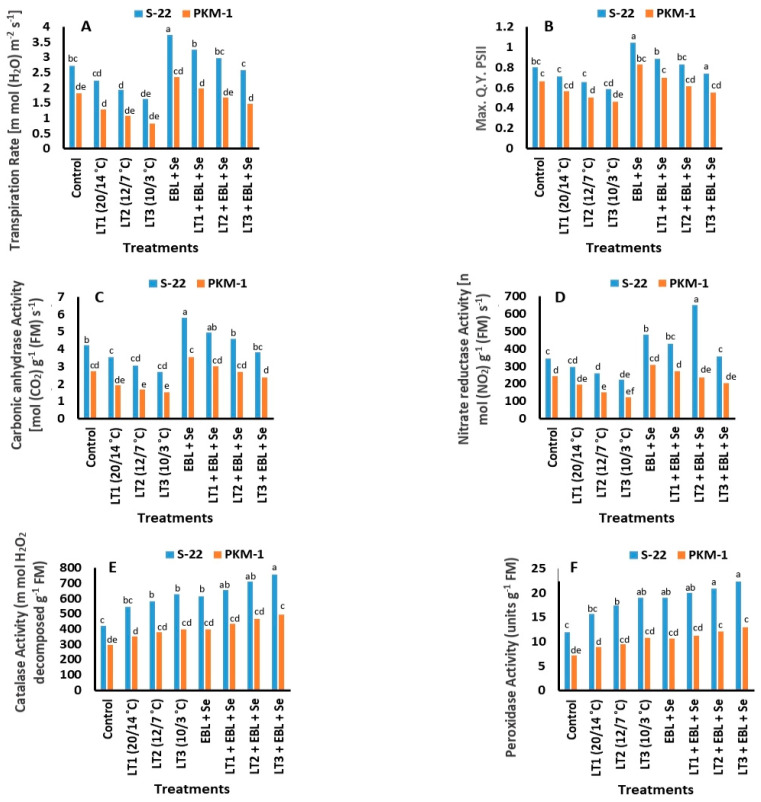
Treatment with EBL and Se altered the (**A**) transpiration rate, (**B**) quantum yield of PS II, (**C**) CA, (**D**) NR, (**E**) catalase (CAT), and (**F**) peroxidase (POX) in the S-22 and PKM-1 varieties of tomato at 60 DAS. All the data are the mean of five replicates (n = 5). Means not sharing the same letters above the bars are significantly different at *p* ≤ 0.05.

The net photosynthetic rate (*P_N_*) and interrelated parameters, including the internal CO_2_ concentration (*Ci*), stomatal conductance (*gs*), and transpiration rate (*E*), increased considerably with the progression of plant growth (Figure 2D–F and Figure 3A). It was observed that the combined treatment of S-22 and PKM-1 plants with EBL + Se enhanced the *P_N_* by 42% and 35%, respectively, the *gs* by 43% and 32%, respectively, the *Ci* by 37% and 28%, respectively, and the *E* by 37% and 29%, respectively, compared to those of the untreated plants. The aforementioned parameters decreased considerably following exposure to low temperatures of 20/14, 12/7, or 10/3 °C at 60 DAS in both the varieties. The damage was more evident in the PKM-1 variety than in S-22 plants. Furthermore, the treatment of S-22 and PKM-1 plants with EBL + Se completely reversed the impairment caused by low temperatures of 20/14 and 12/7 °C. However, the photosynthetic response of the S-22 variety following treatment was superior to that of PKM-1 plants.

### 2.6. Photosystem II (PS II; Fv/Fm)

The Fv/Fm values of S-22 and PKM-1 plants decreased significantly under low-temperature stress at 60 DAS. The reduction was most pronounced in plants exposed to the lowest temperature of 10/3 °C, and the Fv/Fm values of S-22 and PKM-1 plants were lower than those of the respective controls by 28% and 31%, respectively. However, combined treatment with EBL + Se significantly increased the Fv/Fm values of the control plants and plants exposed to low-temperature stress. Additionally, combined treatment with EBL + Se completely reversed the effects of low-temperature stress in S-22 plants.

### 2.7. Activities of Nitrate Reductase (NR) and Carbonic Anhydrase (CA)

The exposure of S-22 and PKM-1 plants to low-temperature stress markedly decreased the activities of CA and NR compared to those of the untreated plants (Figure 3C,D). However, the reduction in enzyme activity was more obvious in the plants exposed to the lowest temperature of 10/3 °C. Compared to those of non-stressed plants, combined treatment with EBL + Se under low temperatures of 20/14, 12/7, or 10/3 °C significantly increased the activity of CA by 25%, 30%, and 36%, respectively, in S-22 plants and by 18%, 24%, and 31%, respectively, in PKM-1 plants at 60 DAS, and the activity of NR increased by 20%, 27%, and 39%, respectively, in S-22 plants and by 15%, 21%, and 28%, respectively, in PKM-1 plants. Furthermore, combined treatment with EBL + Se ameliorated the adverse effects caused by low-temperature stress in S-22 plants.

### 2.8. Activities of Antioxidant Enzymes

**Figure 4:**
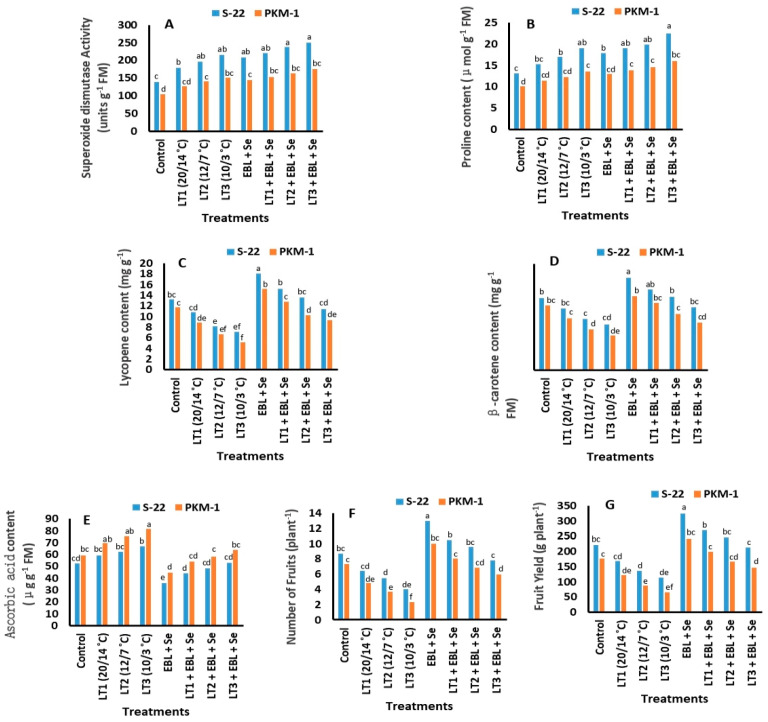
Effect of treatment with EBL and Se on the (**A**) activity of superoxide dismutase (SOD), (**B**) proline content, (**C**) lycopene content, (**D**) β-carotene content, (**E**) ascorbic acid content, (**F**) number of fruits, and (**G**) fruit yield in the S-22 and PKM-1 varieties of tomato exposed to low-temperature stress. All the data are the mean of five replicates (n = 5). Means not sharing the same letters above the bars are significantly different at *p* ≤ 0.05.

The activities of the antioxidant enzymes, catalase (CAT), superoxide dismutase (SOD), and peroxidase (POX), increased following exposure to low temperatures and treatment with EBL and Se. The activities of these antioxidative enzymes were the lowest in the control plants. The activities of CAT, POX, and SOD were highest in S-22 plants grown at 10/3 °C and treated with EBL + Se (Figure 3E,F and Figure 4A), and the activities were 80%, 90%, and 81%, respectively, at 60 DAS.

### 2.9. Proline Content

The proline content of S-22 and PKM-1 plants increased significantly in response to low-temperature stress and was further enhanced by treatment with EBL and Se (Figure 4B). The increase in the proline content was more marked in the S-22 variety than in PKM-1 plants. The proline content of both varieties was highest following exposure to the lowest temperature of 10/3 °C and treatment with EBL + Se at 60 DAS. The proline content of the S-22 and PKM-1 varieties was 73% and 59% higher, respectively, than that of their respective controls.

### 2.10. Contents of Lycopene, Beta-Carotene, and Ascorbic Acid

The contents of lycopene and beta-carotene in the fruits of plants treated with EBL + Se were higher than those of the control plants (Figure 4C,D). The contents of lycopene and beta-carotene were highest in plants treated with EBL + Se, and their contents increased by 36% and 28%, respectively, in S-22 plants, and by 28% and 15%, respectively, in PKM-1 plants, compared to those of the respective controls. The S-22 and PKM-1 plants raised under low-temperature stress contained lower levels of lycopene and beta-carotene, and the levels were markedly reduced at 10/3 °C.

The accumulation of ascorbic acid was highest in the fruits of both varieties at low temperatures of 10/3 °C. However, combined treatment with EBL + Se reduced the content of ascorbic acid, as the treatment of stressed and non-stressed plants decreased the ascorbic acid content compared to that of the control plants (Figure 4E).

### 2.11. Yield and Number of Fruits

Low-temperature stress reduced the number and yield of fruits in both the varieties, compared to those of the respective controls (Figure 4F,G). The damage caused by low-temperature stress was more evident in the PKM-1 variety than in S-22 plants. However, combined treatment with EBL + Se considerably improved the number and yield of fruits per plant, which were highest in S-22 plants treated with EBL + Se under stress-free conditions, being 49% and 24% higher, respectively, than those of the control plants.

### 2.12. Analysis of Protein Expression

A total of 13 differentially expressed proteins were identified by analyzing the protein expression profiles of the tomato cultivars following treatment with EBL + Se. Analysis of the overall expression patterns revealed that the synergistic effect of EBL and Se induced the expression of the highest number of proteins in all the treatment groups. Protein expression was more markedly increased by treatment with EBL + Se than by low-temperature stress. Although the protein expression patterns were also high under low-temperature stress, the sustainability of protein expression was not as high as treatment with EBL + Se. The findings revealed that nine of the 13 differentially expressed proteins were upregulated in the plants exposed to low temperatures of 20/14 °C; however, all the 13 proteins were upregulated following exposure to low temperatures of 10/3 °C. However, only 3 of the 13 proteins, namely, O04939, Q54YH4, and Q9M817, were significantly upregulated in plants exposed to low temperatures of 12/7 °C (Table S1).

**Figure 5:**
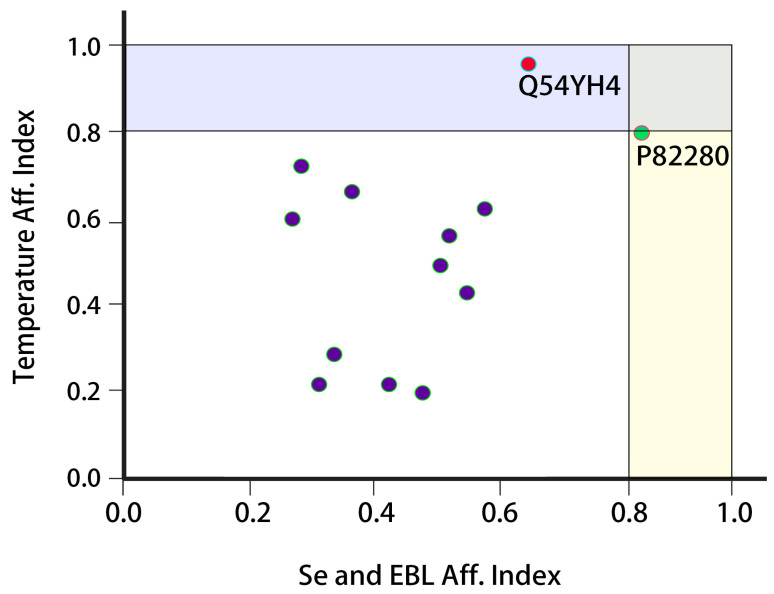
Principal component analysis (PCA) of the S-22 and PKM-1 varieties of tomato. The x-axis represents the affinity index of EBL + Se and the y-axis denotes the low-temperature affinity index. The color points in blue represent the protein species not having a significant correlation with EBL, Se and low temperature stress. However, the color points in green and red represent the key proteins with the highest correlation coefficient values with EBL, Se and low temperature stress.

Functional analyses of the proteins revealed that they play diverse roles in cellular metabolism in plants. The P82280 protein functions as a transcriptional activator of plant cells and partakes in cellular defense by binding to the GCC-box pathogenesis-associated promoter element. P82280 also acts as a regulator of gene expression and functions as a component of signal transduction pathways under stress. The protein acts as a transcriptional repressor during flowering and directly induces the expression of FT by binding to the 5′-CAACA-3′ and 5′-CACCTG-3′ sequences (Probable). P82280 is believed to be functionally like TEM1. The Q54YH4 protein is involved in the cytokinin signal transduction pathway, regulates spore germination during the reproductive stage of plants, and determines spore dormancy. A conserved histidine residue in the kinase core of Q54YH4 undergoes ATP-dependent autophosphorylation, following which the phosphoryl group shifts to a conserved aspartate residue in the receiver domain. The O04567 protein is considered to have ATP binding and kinase activities. The 35336 protein is involved in fruit ripening, and the findings revealed that Q9AXJ4, Q766C3, Q9M817, Q8GXG1, Q04057, O22769, Q94KK7, and Q766C2 were also differentially expressed in the tomato plants (Table S2). The results of principal component analysis (PCA) revealed that only two proteins, namely Q954YH4 and P82280, were strongly correlated with EBL + Se treatment and low-temperature stress. There were variations in the affinity index values of the other 11 proteins, but the variations were lower than those of Q954YH4 and P82280. The affinity index value of P82280 was 0.82 and 0.8 following treatment with Se and exposure to low-temperature stress, respectively. The temperature affinity value (0.94) of Q54YH4 was higher than that of P82280, although its interaction with Se was only marginally stronger (0.63) than that of P82280 (Figure 5).

**Figure 6:**
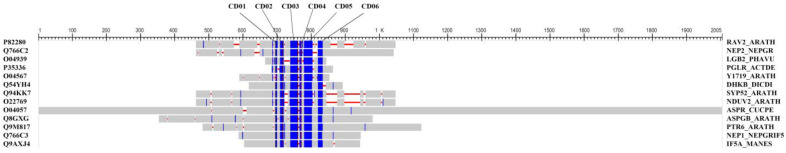
Sequence alignment and conservation analysis of the S-22 and PKM-1 varieties of tomato exposed to low temperatures and subjected to combined treatment with EBL + Se. The specific shared sequences in all the studied proteins are highlighted in blue with specific sequence markings. The left side of each sequence is labeled with the accession number, while strain details of the tested sample are furnished on the right side of each sequence.

The results of sequence alignment and conservation analyses revealed that the degree of sequence homology was higher than 63%. Interestingly, the proteins contained several highly conserved regions that were evenly distributed along the sequences. A total of six major conserved regions were detected in the protein sequences, including regions CD01, CD02, CD03, CD04, CD05, and CD06. CD01 was the smallest conserved sequence with 6 amino acids, and CD05 was the largest conserved sequence with 25 residues. CD03 was the second largest conserved sequence comprising of 22 amino acids (Figure 6).

## 3. Discussion

Plants have developed potent mechanisms of resilience for coping with changing environmental conditions. The mechanism of resilience is determined by signaling cascades that link the internal and external indicators. It has been reported that treatment with BRs and Se under low-temperature stress regulates numerous physiological and biochemical activities in plants, including seed germination, leaf area, chlorophyll synthesis, photosynthesis, production of antioxidants, and chloroplast development [16,17,33,34,43]. However, the mechanism underlying the regulation of these factors by low-temperature stress and combined treatment with BRs and Se remains to be elucidated. The present study revealed that the suppression of growth parameters, including the length of roots and shoots, leaf area, fresh and dry masses of roots and shoots, was more pronounced in PKM-1 plants under low-temperature stress than in the S-22 variety (Figure 1A–G). The findings of the study by Lukatkin et al. [44] revealed that the number of dividing cells and mitotic index of new leaves were reduced under low-temperature stress. The results of the study by Khan et al. [34] revealed that low temperatures significantly reduced the leaf area, SPAD chlorophyll levels, and the fresh and dry weights of the shoots and roots of tomato plants. The findings of the present study revealed that combined treatment with BRs and Se ameliorated the deleterious effects of low-temperature stress. Interestingly, the results demonstrated that low concentrations of Se increased the growth of tomato seedlings, leaf area, and chlorophyll content, and the findings were consistent with the results of earlier studies [16,17,45]. Similar findings have been reported in other plant species, including ryegrass, lettuce, wheat, and *B*. *juncea* [16,17,19,46,47]. Catterou et al. [48] suggested that the treatment of plants with BRs (EBL) has a progressive effect on cell division and elongation by triggering the expression of genes required for the regulation of enzymes such as xyloglucan endotransglucosylases/hydrolases. This consequently mediates the adaptation of cellular development, the movement of the cell wall, and the biosynthesis of cellulose and sucrose [49], which play important roles in plant growth. A study by Bajguz and Tretyn [50] revealed that the foliar application of EBL increased the leaf area and the fresh and dry weights of the roots and shoots of tomato plants, which could be attributed to the increase in cell growth and division. Furthermore, the findings of the present study provided novel insights into the additive outcomes of combined treatment with Se and BRs on the physiological parameters of plants.

In this study, the combined application of EBL and Se significantly increased the SPAD chlorophyll levels (Figure 2A), and the functions of the photosynthetic machinery (Figure 2D–F and Figure 3A) and PS II (Fv/Fm; Figure 3B). However, the present study revealed that low temperatures considerably negatively affected the parameters associated with photosynthesis and declined the SPAD chlorophyll value and productivity of PS II (Figure 3B). It was assumed that the initiation of senescence and the alterations in the functions of interrelated enzymes cause variations in the cytoplasmic composition, modulate the action of sinks, and slow down the transport of photosynthates [51], which in turn reduce the rate of photosynthesis. Other studies have reported comparable variations in photosynthetic efficiency and photosynthesis-related parameters in plants exposed to low-temperature stress [2,34]. The results of the present study are consistent with the findings of Lu et al. [52] in that the *PN* was found to equate with the *gs* and *Ci*. The study by Wu et al. [53] further revealed that all the opposing effects potentially reduce the natural efficacy of the photosynthetic parameters under low temperatures. The present study revealed that the *PN*, *gs*, *Ci*, and *E* improved significantly following treatment with Se under stress or stress-free conditions. However, treatment with Se enhanced the photosynthetic efficiency by modulating the activity of the plant defense mechanism to varying degrees. Treatment with Se increased the content of ascorbate and upregulated the activity of the antioxidant enzymes, SOD, CAT, and POX, which enhanced the ROS scavenging ability of plants [24,54]. Analysis of the effect of Se on the antioxidant mechanism revealed that treatment with Se markedly enhanced photosynthesis by upregulating chlorophyll synthesis [16,17,18] and the photosynthetic attributes (*PN*, *gs*, *Ci*, and *E*) [16,17,18]. A previous study reported that the application of Se under stress improved the leaf area, SPAD chlorophyll levels, photosynthesis-related attributes, and the activity of PS II in *B*. *juncea* [17]. In this study, treatment with EBL enhanced the photosynthetic parameters (*PN*, *gs*, *Ci*, and *E*) which improved CO_2_ assimilation and enhanced the efficacy of the light-harvesting complex by increasing the chlorophyll content. Additionally, Hola et al. [55] revealed that treatment with BRs enhances the progression of net photosynthesis under stress and stress-free conditions. The results of the present study are consistent with the findings of several previous studies which revealed that the exogenous application of BRs enhance photosynthesis, the interrelated features, and the quantum yield of PS II under conditions of stress [2,33,56]. Additionally, Khan et al. [34] recently demonstrated that the treatment of tomato plants with BRs enhanced the photosynthetic parameters (*PN*, *gs*, *Ci*, and *E*) and the activity of PS II (Fv/Fm) under low-temperature stress.

In this study, treatment with EBL and Se increased the proline content in the leaves under low-temperature stress (Figure 4B) and enhanced the activity of the antioxidant enzymes (Figure 3E,F and Figure 4A). However, low temperatures are known to increase the generation of ROS, predominantly in species with lower antioxidant capability, to detoxify ROS, and Se protects plants against oxidative injury under such conditions. A previous study reported that the treatment of cucumber plants with Se reduced the symptoms initiated by salt stress via decreasing the levels of MDA and improving the efficacy of antioxidant enzymes [57]. Comparable findings were observed in potato, cucumber, and sorghum plants treated with Se and exposed to low temperatures [13,58,59]. A recent study demonstrated that compared to those of a sensitive variety, the proline content and mechanism of antioxidant defense in a resistant variety of tomato were enhanced by low temperatures [34]. The study by Yusuf et al. [17] revealed that the treatment of *B. juncea* plants with Se increased the proline content of the leaves and antioxidant activity under copper stress. Another study reported that the increased activity of antioxidant enzymes following treatment with EBL is possibly attributed to the upregulation of det-2, which confers resistance to oxidative damage in Arabidopsis sp. [49]. Treatment with BRs has been shown to enhance the activity of NADPH oxidase accompanied by the accumulation of high concentrations of H_2_O_2_ in apoplasts [60]. BRs are perceived by receptors that activate the membrane-bound NADPH oxidase enzyme, which consequently enhances the H_2_O_2_ content to initiate a protein phosphorylation cascade [61]. The application of EBL under low temperatures enhances oxidative damage in Chorispora bungeana by increasing the content of ascorbate and the activities of CAT, POD, APX, and SOD [61]. It has been reported that the exogenous application of EBL in *B. juncea* seedlings exposed to cold stress reduces the detrimental consequences of H_2_O_2_ by enhancing the activities of various defense-related enzymes, including CAT, SOD, and APX. The study by Khan et al. [34] revealed that the treatment of plants with EBL promoted the activities of CAT, POX, and SOD under low temperatures. The combination of EBL and Se increased plant proline content and the level of antioxidant enzymes, probably through its action at transcription and/or translation levels, protecting the tomato plants from the damage induced by the low-temperature stress.

Plant cells employ various mechanisms for responding to external stimuli. PCA is performed to isolate a specific factor associated with a certain stimulus, based on the frequency and intensity of the plant response. PCA is a very powerful tool that is used to screen the desired (targeted) factor from a densely rich pool of factors. This is easily performed by recording the frequency and intensity of the external stimuli followed by comparison with plant response profiles for calculating the correlation coefficients. The associations of these coefficient values are plotted in scatter plots for determining the principal component from a complicated data pool. The present study employed a holistic approach by recording the affinity index values of the plant proteins following combined treatment with EBL + Se under low temperatures. The proteins that play a key role in the defense responses during treatment with EBL + Se under low-temperature stress were identified by analyzing the differentially expressed proteins. These data were used to screen two major protein species by statistical analysis based on bioactivity-guided assays. The uniqueness of the present study lies in the use of modern data-mining tools, which saved the time required for laborious confirmatory assays. The differentially expressed proteins that are involved in plant metabolism following treatment with EBL + Se under low-temperature stress were identified. Subsequent functional analyses revealed that the Q54YH4 protein plays an active role against plant stressors, thereby confirming the authenticity of the results obtained herein. Structural and sequence analyses of the identified proteins were also performed, and the conserved sequences were analyzed for determining the reliability of plant responses against the external application of EBL + Se under low-temperature stress. The conserved regions were found to be evenly distributed in the protein sequences and had varying lengths, which indicated that the activities of the identified proteins were reliable and stable.

## 4. Materials and Methods

### 4.1. Plant Materials

Seeds of the S-22 and PKM-1 varieties of *Lycopersicon esculentum* were provided by the Department of Horticulture, Indian Agricultural Research Institute New Delhi, India. The seeds were surface-sterilized by incubating them in a 1% sodium hypochlorite solution for 10 min, and then rinsed thrice with deionized water [16].

### 4.2. Preparation of EBL and Se

EBL was procured from Sigma-Aldrich India Ltd. Chemicals, Bangalore, Karnataka, India. A required quantity of the hormone was dissolved in 5 mL of ethanol and placed in a 100 mL volumetric flask to create stock solutions (10^−4^ M) of EBL. The final volume was maintained at the desired level by adding double-distilled water. Sodium selenate (Na_2_SeO_4_) was procured from Sigma-Aldrich India Ltd. Chemicals and was used as the source of Se in this study. Na_2_SeO_4_ was dissolved in deionized water to prepare the stock solution (1 mM), and the final volume was increased to the required level by adding double deionized water. The requisite concentrations (10 μM) of Se were prepared by diluting the stock solution and 24-EBL at a certain concentration based on our earlier study [16]. Prior to the treatments, a desired concentration of 0.5 mL of Tween-20 was applied as a surfactant to all the plants.

### 4.3. Experimental Setup

The seeds were sterilized by incubating them in a 1% sodium hypochlorite solution for 10 min, followed by rinsing thrice with double-distilled water. The sterilized seeds were sown into new earthen pots for creating the nursery. The plants were transplanted under ambient environmental conditions at 20 DAS into earthen pots (25 cm in diameter) filled with sandy-loam soil and farmyard manure (6:1 v:v). The plants were transferred to a plant growth chamber (MAC-MSW-130 Plant Growth Chamber, New Delhi, India) where they were kept for 24 h and grown until 40 DAS under controlled day/night temperatures of 25/18 (control), 20/14, 12/7, or 10/3 °C. A 12 h photoperiod was maintained in the growth chamber using cool white fluorescent (CWF) lights with photosynthetic active radiation (PAR) of 750 ± 50 µmol·m^−2^·s^−1^ and 400 W High-Pressure Sodium (HPS) lamps (Philips Light Company, Lynn, MA, USA). Ambient CO_2_ concentrations were maintained in the growth chamber. The HPS lamps were switched on after 1 h of turning off the CWF lamps each morning and were turned off 1 h before turning on the CWF lamps at night. At 50 DAS, the plants were treated with 10^−8^ M EBL via foliar application, and 10 µM of Se, administered through the soil. The control plants were treated with deionized water. The sprayer nozzles were adjusted to deliver approximately 1 mL of solution per spray. The foliage received 3 mL of EBL solution, and 3 mL of Se solution was added to the soil for three days. Each treatment comprised three plants and the treatment was replicated five times. Several growth factors, photosynthetic parameters, biochemical parameters, and protein expression were evaluated at 60 DAS. The remaining plants in the treatment setups were grown until maturity (approximately 180 DAS) and the ripened fruits were collected for measuring the contents of lycopene, beta-carotene, and ascorbic acid.

### 4.4. Analysis of Plant Growth

The plants were removed from the pots and immersed in a bucket of water. After gently removing the adhering soil, the lengths of the roots and shoots were measured using a meter scale [16]. The area of the leaves was measured using a leaf area meter (ADC Bioscientific, Hoddesdon, UK).

### 4.5. Physiological Analysis

The maximal quantum yield of PS II, gas exchange, chlorophyll content (SPAD value), and LWP were measured as described in our previous study [2].

### 4.6. Biochemical Analysis

The activities of CA, NR, CAT, POX, and SOD and the accumulation of proline, lycopene, beta-carotene, and ascorbic acid were determined as described in our previous study [2].

### 4.7. Analyses of Protein Expression

The total protein profile was analyzed for identifying the proteins that were differentially expressed under low-temperature stress. The total protein was extracted according to the method described by Ahmad et al. [42]. Briefly, 140 mM NaCl, 10 mM Na_2_HPO_4_, 1.8 mM NaH_2_PO_4_, and 2.5 mM KCl were used as extraction solvents (sodium phosphate-buffered saline; pH 6.8). The protein samples were dissolved in 8 M urea solution before native 2D gel electrophoresis. The extracted protein samples were separated using 12% gels by native polyacrylamide gel electrophoresis (PAGE). Sodium dodecyl sulfate was added during the second-dimension electrophoresis step for enhancing the resolution. Coomassie blue staining was performed for visualization and documentation of the results. Digital images were captured for detailed analyses of the protein gels.

### 4.8. Identification and Image Analysis

The protein expression profiles of each cultivar were compared to those of the corresponding negative controls for calculating the profusion index for each protein using the formula previously described by Khan et al. [34]. Briefly, the profusion behavior of several proteins was plotted in a matrix plot and sorted based on their profusion indices. The proteins that were most actively involved in the response against environmental stress and exogenous hormones were identified based on the profusion index. The profusion index was calculated using the following formula:

Profusion Index = ΣInd/Frequency of occurrence

where ΣInd represents the number of times a protein was expressed in response to a stimulus, compared to that in the control.

The identified protein spots were analyzed and compared using the SAMESPOTS (Total Lab Ltd., Newcastle, UK) and TOPSPOT (Kroger and Prehm, Berlin, Germany) digital software. The characteristics of each protein were determined from the UniProt database. The physical properties of the proteins were precisely determined by solution-state NMR spectroscopy. The NMR data were statistically analyzed and compared to existing data in the online protein databases using the PSVS software (Northeast Structural Genomics, NESG). This step was performed to further verify the protein identification, as it was screened and identified by the combined application of image and statistical analysis.

### 4.9. Identification of Protein Function

Functional analysis of the proteins revealed that their involvement in cellular physiological processes was strongly associated with cold stress and the application of hormonal treatments. For functional analysis, the translation of three target proteins was ceased in different plant samples using a specific short inhibitory RNA. To terminate the protein expression, a two-step process was performed consisting of the determination of conserved sequence in the protein sequence. It generated the conserved intervals of 4-144_Globin-like for O04939, 668-818_Acyltransferase for Q54YH4, and 28-566_ NRT1/PTR for Q9M817. Then, the sequences underwent a BLAST run with the other genome sequences available in the database, and the inhibitory sequences were constructed with the least sharing intervals with other proteins to minimize the impact of inhibitory RNA on the expression of other proteins in the cell. Therefore, the inhibitory sequences obtained were 84–102 for O04939, 668–687 for Q54YH4, and 32–50 for Q9M817. Then, following translational termination, the total metabolites were collected and examined by gas chromatography mass spectrometry (GCMS) using a Clarus SQ8 system (Perkin Elmer, Shelton, CT, USA), according to the protocol described in our previous study [42]. The plant extracts were diluted at room temperature in methoxamine (MOX) reagent and incubated overnight. Then, 20 L of N-trimethylsilyl N-trifluoroacetamide (MSTFA) was separately added to each extract and the mixture was incubated for 30 min before loading 3 L of the mixture into the GCMS apparatus. The initial oven temperature was maintained at 70 °C and subsequently increased continually at a rate of 5 °C min^−1^ until it reached a temperature of 200 °C. The temperature was further raised to 240 °C at a constant rate of 10 °C min^−1^. The obtained mass spectra were analyzed using the MZ mine software (Pluskal, Okinawa, Japan). The changes in the cellular metabolites were mapped using a metabolic pathway that reflects the pathways of sugar and amino acid metabolism in plant cells. A metabolic route accurately depicting the energy flow and balance of metabolic processes was constructed using this method.

### 4.10. Statistical Analyses

Each treatment was performed in triplicate and the mean values of different factors in the different treatment groups were compared using SPSS version 17 (Chicago, IL, USA).

## 5. Conclusions

Of the abiotic stresses, low-temperature stress poses a predominantly severe challenge to horticulture crops and significantly limits their productivity. The present study revealed that the exposure of two varieties of tomato plants to low temperatures significantly reduced the values of growth biomarkers, SPAD chlorophyll levels, photosynthesis-related attributes, and activity of NR in both varieties. The combined application of EBL and Se significantly improved the aforementioned metrics under conditions of stress and non-stress. However, exposure to low temperatures increased the activities of the antioxidative enzymes (POX, CAT, and SOD) and the proline content of leaves, which were further enhanced by combined treatment with Se and EBL in both varieties. Moreover, modern data mining tools were used to determine the differentially expressed proteins that were involved in plant metabolism after treatment with EBL + Se at low temperatures. It was discovered through functional analysis of the differentially expressed proteins that the Q54YH4 protein actively defends plants from stresses. The conserved regions in the protein sequences were further analyzed for determining the reliability of plant responses against the external application of EBL + Se under low temperatures.

## Data Availability

The data supporting the findings of this study are available within the article.

## Supplementary Materials

The following supporting information can be downloaded at: https://www.mdpi.com/article/10.3390/plants12193351/s1, Table S1. Relative abundance of protein species quantified in two tomato cultivars, (S-22 and PKM-1) treated with combinations of EBL plus Se and low temperature. Up-regulations and down-regulations of protein expressions are indicated by arrow sign. Table S2. Three dimensional models of most active proteins in two tomato cultivars, (S-22 and PKM-1) treated with combinations of EBL plus Se and low temperature.
