# Supplementary material for: Exploring the Effects of Selenium and Brassinosteroids on Photosynthesis and Protein Expression Patterns in Tomato Plants under Low Temperatures
Source: Plants (Basel). 2023 Sep 22;12(19):3351. doi: 10.3390/plants12193351 (PMC10574566; doi:10.3390/plants12193351)
Supplement: Supplementary file 1 [file plants-12-03351-s001.zip › plants-2624091-supplementary.pdf]

Table S1. Relative abundance of protein species quantified in two tomato cultivars, (S-22 and PKM-1) treated with combinations of EBL plus Se and Low temperature. Up-regulations and down-regulations of protein expressions are indicated by arrow sign.

S. No.	Uniprot ID	LT1 (20/14 ˚C)	LT2 (12/7 ˚C)	LT3 (10/3 ˚C)	EBL + Se	LT1+ EBL + Se	LT2+ EBL + Se	LT3+ EBL + Se
1	P82280	↓	↓	↘	↑	↑	↑	↑
2	Q766C2	↓	↓	↘	→	↘	→	↘
3	O04939	↓	↓	↓	→	→	→	→
4	P35336	↓	↓	↓	→	→	→	→
5	O04567	↓	↓	↓	→	→	→	→
6	Q54YH4	→	↗	↗	↗	↑	↗	↑
7	Q94KK7	↓	↓	↓	→	→	→	→
8	O22769	↓	↓	↘	→	→	→	→
9	O04057	↘	↓	↘	↗	→	↗	→
10	Q8GXG1	↘	↓	↘	↗	→	↗	→
11	Q9M817	↘	↓	↘	→	→	→	→
12	Q766C3	↘	↓	↘	↗	→	↗	→
13	Q9AXJ4	↓	↓	↘	↗	→	↗	↗

Table S2. Three dimensional models of most active proteins (13) in two tomato cultivars, (S-22 and PKM-1) treated with combinations of EBL plus Se and Low temperature.

S. No.	Uniprot ID	Alternative	Function	Structure
1	P82280	RAV2_ARATH	The proteinacts as a transcriptional activator. It binds to the GCC-box pathogenesis-related promoter element. The protein species is involved in the regulation of gene expression by stress factors and by components of stress signal transduction pathways (By similarity). It is also conceptualized as transcriptional repressor of flowering time on long day plants. Acts directly on FT expression by binding 5'-CAACA-3' and 5'-CACCTG-3 sequences (Probable). Functionally redundant with TEM1.	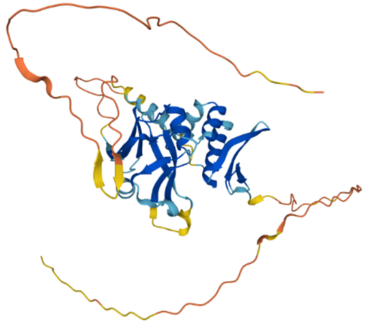
2	Q766C2	NEP2_NEPGR	The protein is famous as extracellular proteinase found in the pitcher fluid of carnivorous plants. It is involved in the digestion of prey for nitrogen uptake and performs catalytic activity just similar to pepsin.	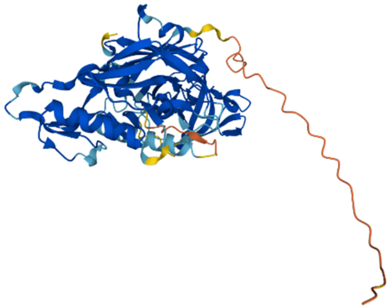

3	O04939	LGB2_PHAVU	The protein species provides oxygen to the bacteroids. This role is essential for symbiotic nitrogen fixation.	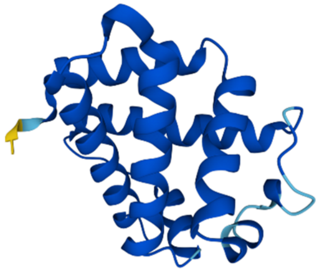
4	P35336	PGLR_ACTDE	Protein species acts in concert with the pectinesterase, in the ripening process. It is also involved in cell wall metabolism, specifically in polyuronide degradation.	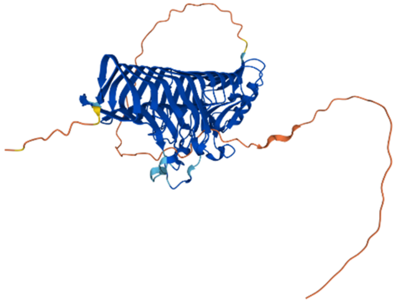
5	O04567	Y1719_ARATH	The protein species is involved in ATP binding and performs protein kinase activity.	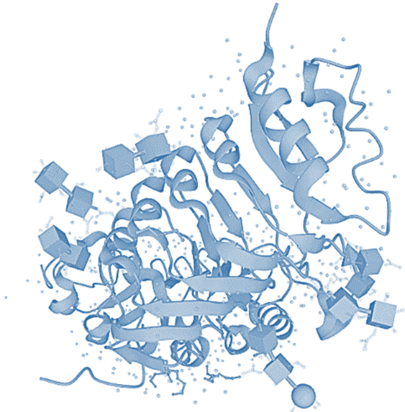

6	Q54YH4	DHKB_DICDI	The protein species acts in the cytokinin signal transduction pathway that regulates spore germination. It is required for the maintenance of spore dormancy. The protein does not appear to act as a cytokinin receptor. It undergoes ATP-dependent autophosphorylation at a conserved histidine residue in the kinase core, which is followed by transfer of the phosphoryl group to a conserved aspartate residue in the receiver domain.	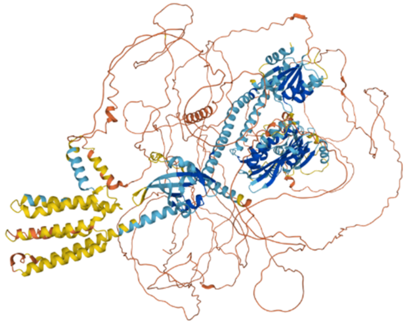
7	Q94KK7	SYP52_ARATH	It is known as vesicle trafficking protein that functions in the secretory pathway.	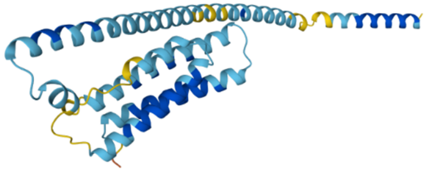

8	O22769	NDUV2_ARATH	Core subunit of the mitochondrial membrane respiratory chain NADH dehydrogenase (Complex I) that is believed to belong to the minimal assembly required for catalysis. Complex I functions in the transfer of electrons from NADH to the respiratory chain. The immediate electron acceptor for the enzyme is believed to be ubiquinone (By similarity).	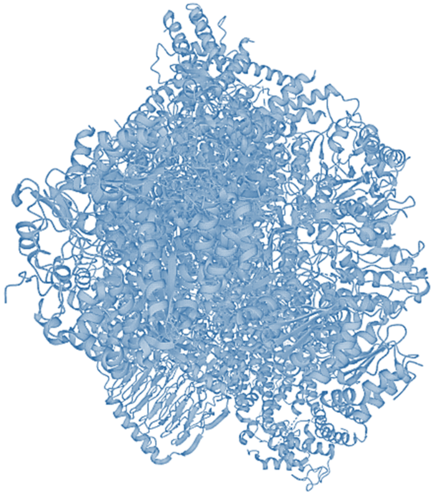
9	O04057	ASPR_CUCPE	Involved in the breakdown of propeptides of storage proteins in protein-storage vacuoles.	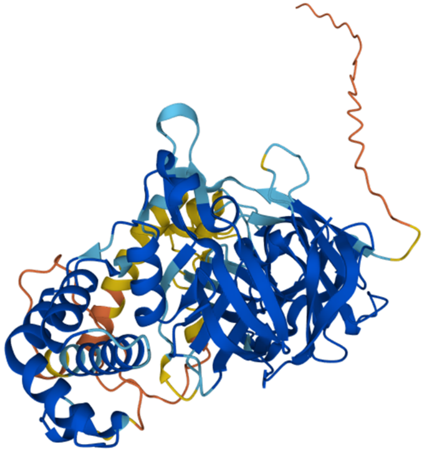

10	Q8GXG1	ASPGB_ARATH	Acts in asparagine catabolism and also in the final steps of protein degradation via hydrolysis of a range of isoaspartyl dipeptides.	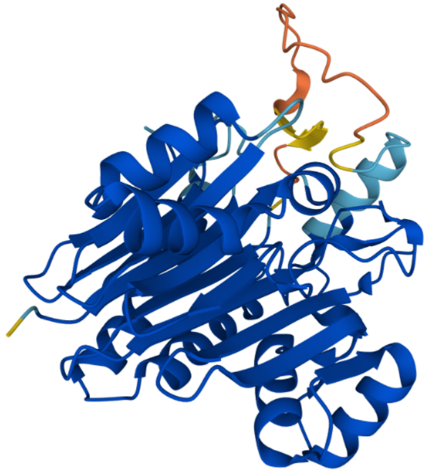
11	Q9M817	PTR6_ARATH	Low-affinity nitrate transporter involved in xylem-to-phloem transfer for redistributing nitrate into developing leaves. Not involved in dipeptides transport.	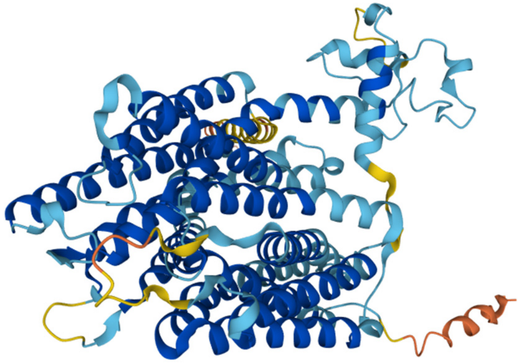

12	Q766C3	NEP1_NEPGR	Extracellular proteinase found in the pitcher fluid of carnivorous plants. Digest prey for nitrogen uptake.	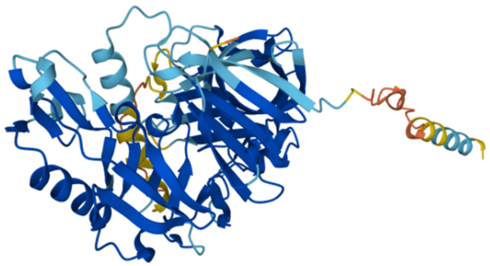
13	Q9AXJ4	IF5A_MANES	The precise role of eIF-5A in protein biosynthesis is not known but it functions by promoting the formation of the first peptide bond.	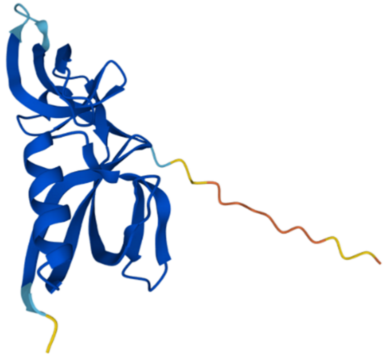
